# Tissue expander placement and adjuvant radiotherapy after surgical resection of retroperitoneal liposarcoma offers improved local control

**DOI:** 10.1097/MD.0000000000004435

**Published:** 2016-08-12

**Authors:** Hyojun Park, Sanghoon Lee, BoKyong Kim, Do Hoon Lim, Yoon-La Choi, Gyu Seong Choi, Jong Man Kim, Jae Berm Park, Choon Hyuck David Kwon, Jae-Won Joh, Sung Joo Kim

**Affiliations:** a Department of Surgery, Samsung Medical Center, Sungkyunkwan University School of Medicine, Seoul, Korea; b Department of Radiation Oncology, Seoul National University Hospital, Seoul, Korea & Sheikh Khalifa Specialty Hospital, Ras al Khaimah, U.A.E.; c Department of Radiation Oncology; d Department of Pathology, Samsung Medical Center, Sungkyunkwan University School of Medicine, Seoul, Korea.

**Keywords:** liposarcoma, radiotherapy, retroperitoneal space, tissue expansion devices

## Abstract

Given that retroperitoneal liposarcoma (LPS) is extremely difficult to completely resect, and has a relatively high rate of recurrence, radiotherapy (RT) is the treatment of choice after surgical resection. However, it is difficult to obtain a sufficient radiation field because of the close proximity of surrounding organs. We introduce the use of tissue expanders (TEs) after LPS resection in an attempt to secure a sufficient radiation field and to improve recurrence-free survival.

This study is a retrospective review of 53 patients who underwent surgical resection of LPS at Samsung Medical Center between January 1, 2005, and December 31, 2012, and had no residual tumor detected 2 months postoperatively. The median follow-up period was 38.9 months.

Patients were divided into 3 groups. Those in group 1 (n = 17) had TE inserted and received postoperative RT. The patients in group 2 (n = 9) did not have TE inserted and received postoperative RT. Finally, those in group 3 (n = 27) did not receive postoperative RT. Multivariate analysis was performed to identify the risk factors associated with recurrence-free survival within 3 years. Younger age, history of LPS treatment, and RT after TE insertion (group 1 vs group 2 or 3) were significantly favorable factors influencing 3-year recurrence-free survival.

TE insertion after LPS resection is associated with increased 3-year recurrence-free survival, most likely because it allows effective delivery of postoperative RT.

## 1 Introduction

Retroperitoneal liposarcomas (LPS) are relatively rare tumors with an annual incidence of 2.5 per million population. ^[1]^ Complete surgical resection is the only known cure for retroperitoneal liposarcoma. However, LPS is often asymptomatic until it becomes a very large retroperitoneal mass. The rate of complete resection is reported to be 40% to 60%.[ ^2^ ^3^] Prior reports have suggested that postoperative radiotherapy (RT) may reduce local recurrence. ^[4]^ However, RT can also have serious complications in these patients. ^[5]^ In this study, we present our experience using tissue expanders (TEs) after complete LPS surgical resection in order to optimize postoperative RT.

## 2 Methods

**Figure 1:**
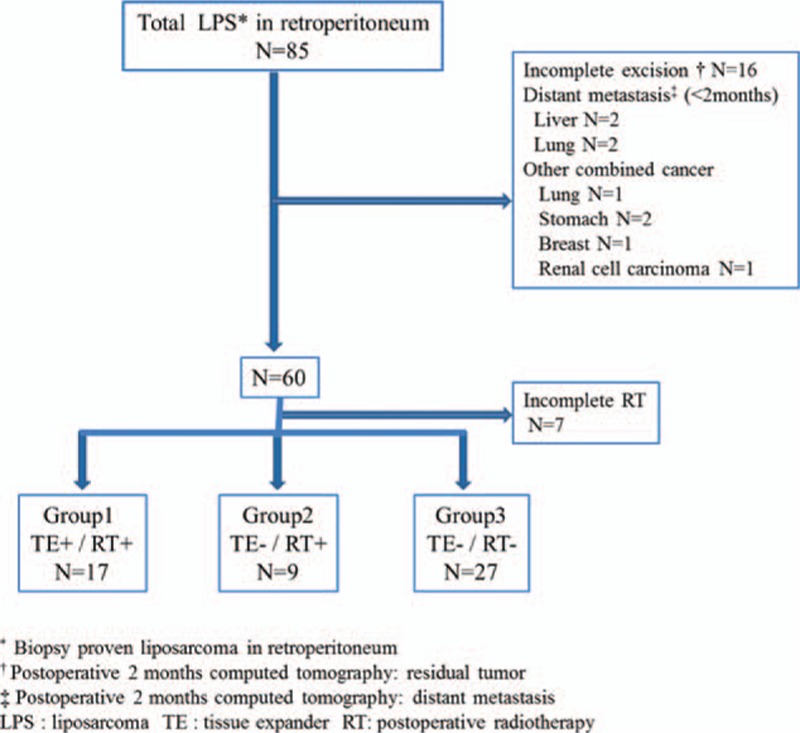
A schematic of patient enrolment. LPS = liposarcoma, RT = postoperative radiotherapy, TE = tissue expander.

A retrospective chart review was performed to identify patients that had been diagnosed with retroperitoneal liposarcoma between January 2005 and December 2012 at Samsung Medical Center. Eighty-five patients underwent surgical resections of LPS. Sixteen patients were excluded because residual tumor was identified on the 2-month postoperative computed tomography (CT) scans (incomplete resection). A complete resection was defined by the absence of any radiologic evidence of residual tumor on the first postoperative CT scan (between 1 and 8 weeks following surgery). Two patients were also excluded because they had liver or lung metastases identified on postoperative CT scan. Six patients were excluded because they had a history of other malignancies diagnosed before liposarcoma resection. Finally, 7 patients were excluded for failure to complete the scheduled radiation therapy. The remaining 53 patients were included in the analyses and were divided into 3 groups: patients who had TE inserted postoperatively and received RT (Group 1, n = 17); patients who received RT without TE insertion (Group 2, n = 9); and patients who did not receive RT (Group 3, n = 27, Fig. 1). The median follow-up period was 38.9 months.

**Figure 2:**
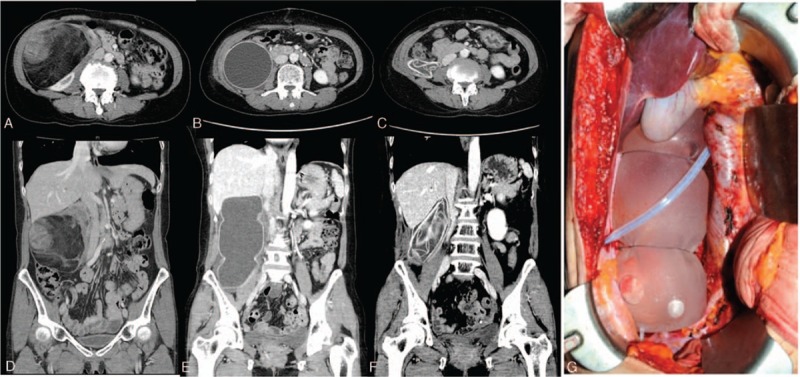
Representative computed tomography (CT) and intraoperative findings. A, Preoperative CT axial image. B, Postoperative (1 wk) CT axial image. C, Postoperative (2 yr 5 mo) CT axial image. D, Preoperative CT coronal image. E, Postoperative (1 wk) CT coronal image. F, Postoperative (2 yr 5 mo) CT coronal image. G, Intraoperative TE insertion finding. A 53-year-old woman underwent a mass excision with right nephrectomy of a dedifferentiated liposarcoma (FNCLCC grade 2/3). A tissue expander (760 mL) with injection valve was inserted and the patient was treated with postoperative radiotherapy (54 Gy). There was no evidence of tumor recurrence after 2 years and 5 months.

The surgical methods and postoperative follow-up have been described previously. ^[6]^ Briefly, primary en bloc tumor resection was performed in all patients. When the tumor was in close proximity to the kidney, the perirenal adipose tissue was removed through ipsilateral nephrectomy. The decision to insert TEs is made during surgery by the surgeon when the liposarcoma is suspected to be dedifferentiated type and will require adjuvant RT. In patients receiving adjuvant RT, 1 to 2 TEs (Tissue Expander LSRT 83 and 82 series; SEBBIN, Boissy L’Aillerie, France) were placed in the tumor bed after resection. Two patients who underwent concomitant bowel resection developed delayed TE infections. Therefore, after this was recognized, TEs were not inserted in patients undergoing bowel resection. TEs were filled with saline. After completion of RT, the saline was drained (and the TEs were deflated) through a subcutaneous port (Fig. 2). Patients were followed with abdominal CTs every 3 to 6 months. If local recurrence was suspected, positron emission tomography-CT (PET-CT) was used to confirm the presence of distant metastases. Whenever possible, recurrent tumors were resected surgically.

Postoperative RT was delivered using 3-dimensional conformal RT (3DCRT) technique. For RT, CT images were taken using CT simulator, and then target and normal organs were delineated by the radiation oncologist. Total dose of 45 to 54 Gy was delivered using 1.8 to 2.0 Gy fraction size considering dose constraint, especially for small bowel.

All adverse events in patients given RT were categorized and graded according to Common Toxicity Criteria for Adverse Events (CTCAE) grade in gastrointestinal contents.

The Kruskal–Wallis and Fisher exact tests were used to analyze continuous and categorical variables, respectively. The Wilcoxon rank sum test was used for continuous variables between the 2 groups (RT dose analyses). Survival curves were compared using the log rank test. Logistic regression analysis was used to analyze the risk of recurrence. *P* values and 95% confidence interval (95% CI) were corrected by Bonferroni method in case of multiple testing. This research has been approved by the institutional review board at Samsung Medical Center (Seoul, Korea).

## 3 Results

### 3.1 Patient characteristics and recurrence patterns

The median patient ages in groups 1, 2, and 3 were 52, 57, and 56, respectively. There was no significant difference in the number of patients who received their first surgery at our center between the 3 groups: group 1 (35%), group 2 (22%), and group 3 (33.3%). Other variables, including the liposarcoma subtype and extent of surgery (nephrectomy or bowel resection), were also not significantly different among the 3 groups. Evaluation of pathologic resection margin was not feasible in 64.7% of group 1, 33% of group 2, and 48.1% of group 3. However, all patients enrolled in the study were confirmed to have no residual tumor on CT taken at postoperative 8 weeks. There were fewer bowel resections in group 1 than there were in groups 2 and 3. This reflects our decision to refrain from TE insertion when concomitant bowel resection was performed. The mean tumor size was significantly larger in group 1 (30 cm) than it was in group 2 (12 cm) and group 3 (27 cm, *P* *=* 0.037, Table 1).

**Table 1:**
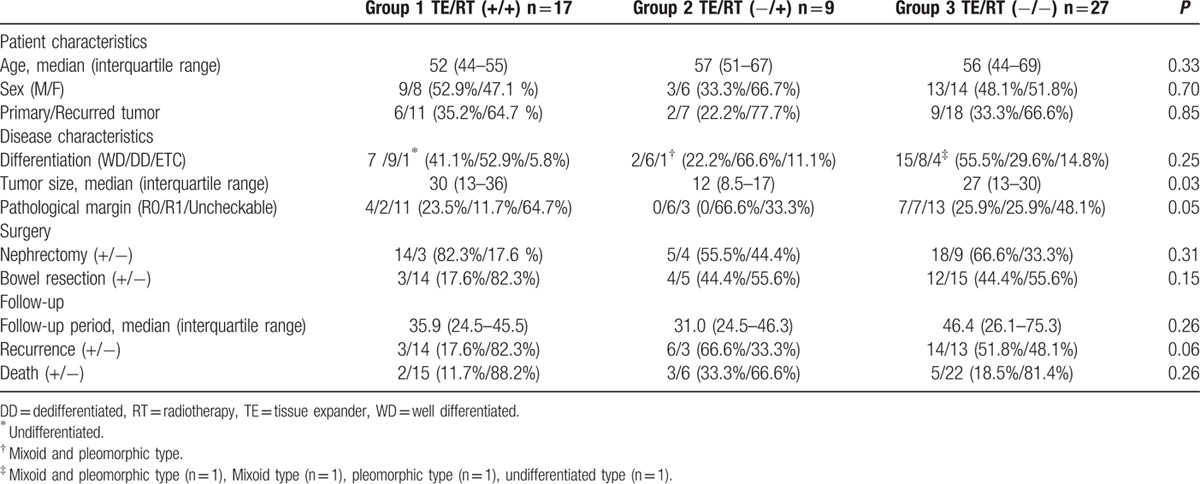
Patient characteristics.

Table 2 summarizes the characteristics of recurrent cases of retroperitoneal liposarcoma. All of the recurrences in group 1 occurred locally, in the abdomen. All of the recurrences in group 2 were in close proximity to the original tumor, except that in patient 4. Fourteen recurrent cases in group 3 were local recurrences. The median time to recurrence was 17 months following resection.

**Table 2:**
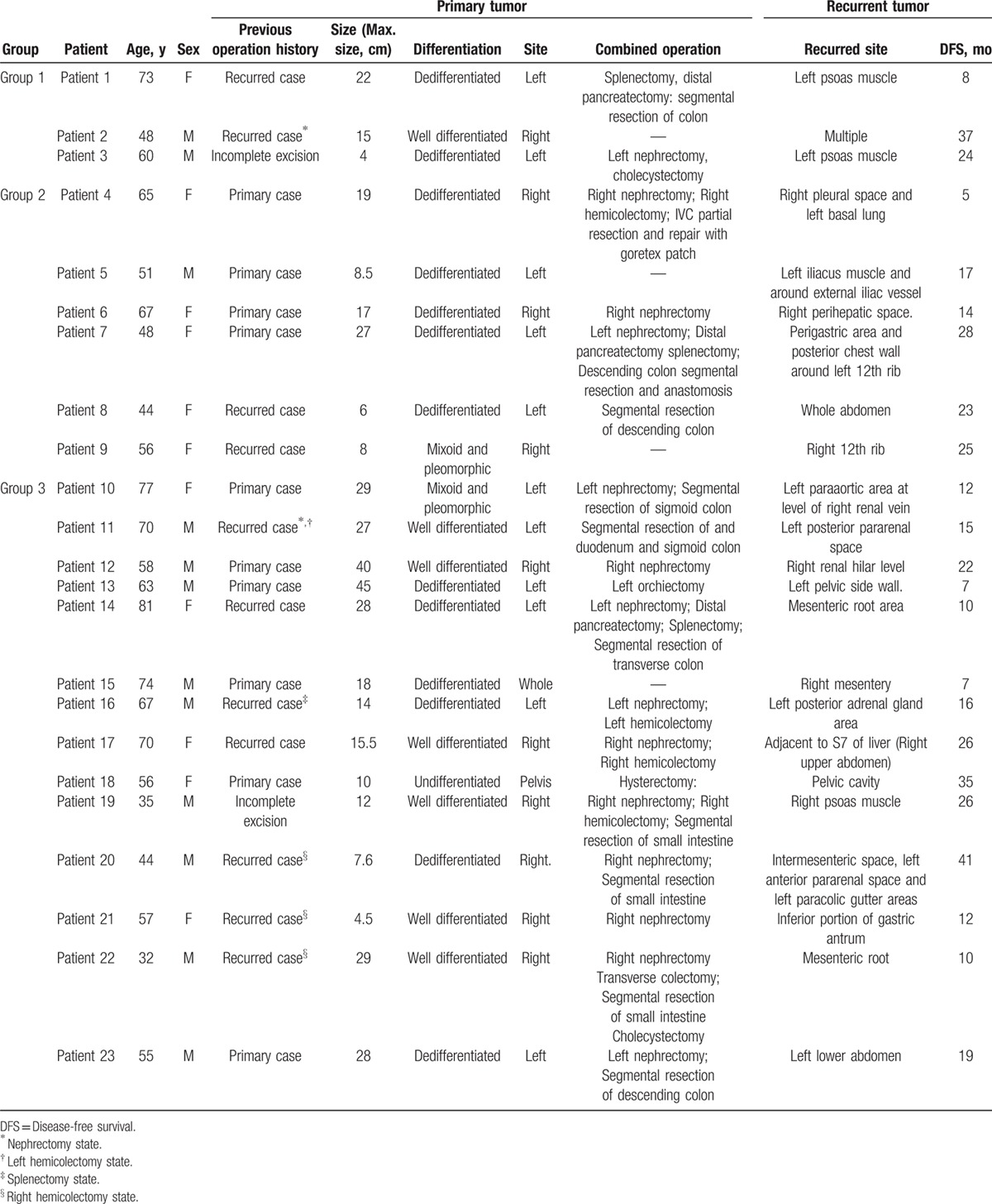
Characteristics of patients with recurrent retroperitoneal liposarcoma.

### 3.2 Pros and cons of tissue expander placement

Six of the nine patients who received RT without TE insertion (group 2) experienced moderate abdominal pain, nausea, and vomiting (CTCAE grade 2). One patient could not complete the RT because of bowel herniation and loss of RT field. One patient had signs, symptoms, and CT evidence of radiation enteritis requiring medical treatment (CTCAE grade 3). In addition to 13 of the 17 patients who received RT with postoperative TE insertion (group 1) had moderate abdominal discomfort and nausea (12 patients as CTCAE grade 2 and 1 patient as CTCAE grade 3) with high radiation doses. There was no significant difference in adverse events after RT between Groups 1 and 2 according to CTCAE classification (*P* = 0.864).

There were complications related to TE insertion in 4 patients. Two patients developed abscesses around the TE following RT. In these patients, the TE had to be removed. The TE was malpositioned in 1 patient, necessitating its removal. Finally, the TE was removed in another patient because of recurrent ileus and abdominal discomfort.

### 3.3 Recurrence-free survival and risk factors for recurrence

**Figure 3:**
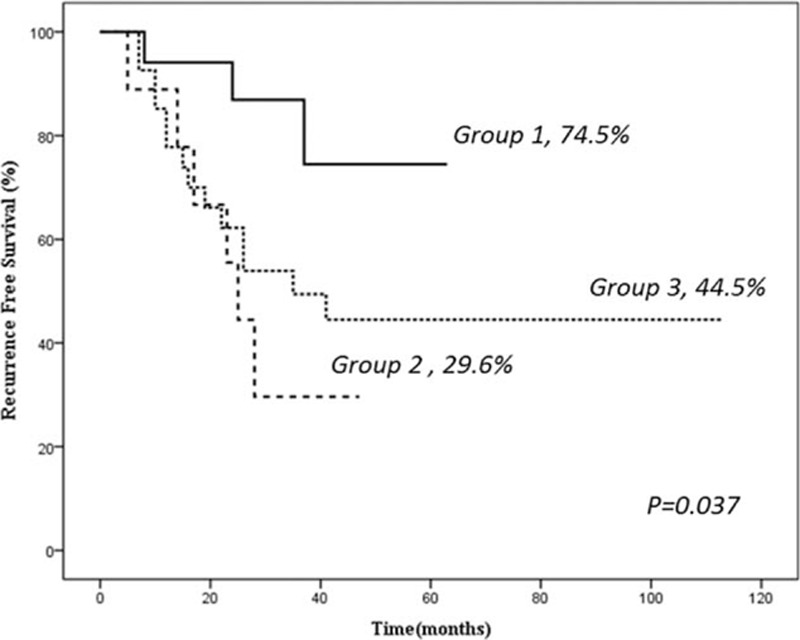
Recurrence-free survival after the surgical resection of retroperitoneal liposarcoma.

There were 3 cases of recurrence in group 1 (17.6%), 6 in group 2 (66.6%), and 14 in group 3 (51.8%, *P* *=* 0.06). Two patients died (11.7%) in group 1, 3 (33.3%) in group 2, and 5 (18.5%) in group 3 (*P* *>* 0.05) (Table 1). Disease-free survival rates at 1, 3, and 5 years were 94%, 86%, and 74% in group 1, 88%, 29%, and 29% in group 2, and 85%, 49%, and 44% (*P* *=* 0.037) in group 3, respectively (Fig. 3).

The risk factors for recurrence were analyzed after 3 years. Univariate analysis demonstrated that group 2 and 3, older age, previous history of liposarcoma treatment, bowel resection (performed during liposarcoma excision) and R1 pathologic margin were significant risk factors for recurrence. Multivariate analysis of these significant risk factors revealed that in Group 2 and 3, previous history of liposarcoma treatment were independent risk factors for recurrence at 3 postoperative years (Table 3).The median RT dose was 54 Gy in group 1 and 50.4 Gy in group 2 (*P* *>* 0.05).

**Table 3:**
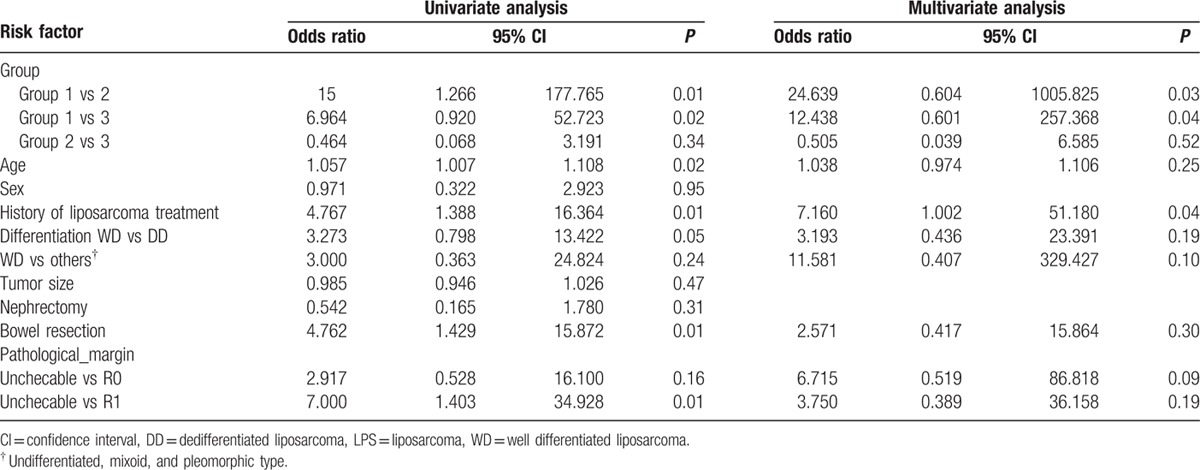
Univariate and multivariate analysis of recurrence risk factors at 3 years.

## 4 Discussion

Soft tissue sarcomas comprise approximately 1% of all solid malignancies. Between 15% and 20% of them occur in the retroperitoneal space. ^[7]^ LPS are the most common type of retroperitoneal soft tissue sarcomas. ^[8]^ The only curative treatment for retroperitoneal liposarcoma is complete surgical resection. [^9^ ^10^ ^11^ ^12^] However, R0 resection rates are only approximately 50%.[ ^10^ ^13^] The low rate of complete R0 resection is partly due to difficulty in thoroughly evaluating negative resection margins in a large pathologic specimen involving multiple organ structures. Recurrence is most often identified 6 to 24 months after surgery. Recurrence is usually locoregional, rather than distant. ^[14]^ We previously reported that 90% of retroperitoneal liposarcoma recurrence occurs within 2 years of surgery. ^[14]^ The median survival of patients with disease recurrence is 27 months. ^[15]^ Surgical resection is the primary method for locoregional control of liposarcoma. However, in the case of multifocal locoregional disease, there is a possibility of remote or outside-field recurrence. ^[16]^ There are also reports of recurrent tumors arising in the residual peritumoral fat. ^[16]^

Prior reports have suggested that postoperative RT effectively controls locoregional recurrence after complete liposarcoma resection.[ ^17^ ^18^] In 1 study, there was a 23% 5-year recurrence-free survival rate in patients with retroperitoneal sarcoma who had surgical resection alone; in contrast, those who also underwent postoperative RT had a survival rate of 55%. ^[19]^ Zhou et al ^[17]^ found that RT was effective in improving survival in patients with abdominal sarcoma and locoregional disease. In this study, recurrence was locoregional in all but 4 cases. There was tumor recurrence in 51.8% of patients who did not receive postoperative RT (group 3). In contrast, there was 66.6% recurrence in group 2 patients who received postoperative RT without TE insertion. Finally, the recurrence rate of patients in group 1 (who received postoperative RT with TE insertion) was 17.6%. The difference in the median RT dose between groups 1 and 2 was not statistically significant. Regardless, the presence of TE seems to have led to more effective radiation delivery and a significant decrease in local recurrence.

An important issue in postoperative RT for retroperitoneal liposarcoma is that the intestines tend to relocate to the original tumor site after surgical removal. This phenomenon makes the intestines vulnerable to radiation. ^[18]^ The incidence of acute enteritis is as high as 80%. ^[4]^ The metabolically active intestinal epithelium is subjected to the cytotoxic effects of radiation, and radiation enteritis subsequently presents with diarrhea. ^[5]^ Chronic radiation enteritis is caused by obliterative endarteritis and leads to tissue ischemia and submucosal fibrosis. These changes ultimately worsen the ischemia and can result in stricture, fistula formation, or intestinal perforation. ^[20]^ Pawlik et al ^[21]^ recommend that the radiosensitive viscera are protected from radiation exposure, irrespective of radiation timing. A TE prevents the bowel from entering the radiation field. Therefore, it may decrease the incidence of bowel-related radiation injury. In this study, most patients who received RT without TE insertion (7 of 9 patients, group 2) had moderate degrees of abdominal symptoms and those who received RT with postoperative TE insertion (13 of 17 patients, group 1) had moderate abdominal discomfort. But 1 patient in group 2 could not complete the RT because of bowel herniation and loss of RT field and another patient in group 2 had experienced radiation enteritis requiring medical treatment.

We found that patients with TE insertion and RT after retroperitoneal liposarcoma resection had superior disease-free survival than did those who did not have TE insertion. Therefore, TE appears to improve survival, in part because it allows for effective radiation delivery with fewer bowel complications. The effects of TE insertion before postoperative RT have similarly been reported for advanced colorectal cancer ^[22]^ and gynecological cancer. ^[23]^ However, there are only sporadic case reports that address the use of TE after retroperitoneal sarcoma resection. [^24^ ^25^ ^26^] Considering that the median follow-up time was 38.9 months, and 90% of recurrences occur within 2 years of surgery, we analyzed the risk factors for 3-year recurrence. ^[14]^ Three years after complete resection, postoperative RT without TE insertion (vs postoperative RT with TE insertion) was an independent risk factor for liposarcoma recurrence.

This study has several limitations. The differences in RT doses between the groups of patients with and without TE insertion were not identified. However, the TE insertion group received higher (albeit not statistically significant) radiation doses than did the group without TE insertion. TE insertion also allowed for more secure RT fields, fewer severe complications, and possibly improved disease-free survival. We have since treated more patients with this protocol and recently increased the RT dose to over 60 Gy. In the future, further analyses are required to assess the long-term effects of these findings. Another limitation of this study is that there was a higher rate of bowel resections in group 2 than that in other groups. TEs were not inserted in cases with concomitant bowel resection for reasons previously explained. However, there were more bowel resections in group 2 than in group 1, which may indicate that group 2 patients had more progressed disease than did those in group 1. Regardless, the fact that group 1 had a lower recurrence rate with significantly larger tumors than those of group 2 may support our hypothesis.

Patients receiving postoperative RT after surgical resection of retroperitoneal liposarcoma with TE insertion have improved 3-year disease-free survival compared with patients either not receiving postoperative RT or not undergoing TE insertion. This finding may be related to TE insertion and the resulting improved RT fields, and the ability to increase the RT dose without adversely affecting adjacent organs.

## References

[1] Alvarenga JC Ball AB Fisher C Limitations of surgery in the treatment of retroperitoneal sarcoma. *Br J Surg* 1991; 78:912–916. 1913104 10.1002/bjs.1800780806

[2] Gronchi A Lo Vullo S Fiore M Aggressive surgical policies in a retrospectively reviewed single-institution case series of retroperitoneal soft tissue sarcoma patients. *J Clin Oncol* 2009; 27:24–30. 19047283 10.1200/JCO.2008.17.8871

[3] Erzen D Sencar M Novak J Retroperitoneal sarcoma: 25 years of experience with aggressive surgical treatment at the Institute of Oncology, Ljubljana. *J Surg Oncol* 2005; 91:1–9. 15999353 10.1002/jso.20265

[4] Zlotecki RA Katz TS Morris CG Adjuvant radiation therapy for resectable retroperitoneal soft tissue sarcoma: the University of Florida experience. *Am J Clin Oncol* 2005; 28:310–316. 15923806 10.1097/01.coc.0000158441.96455.31

[5] Harb AH Abou Fadel C Sharara AI Radiation enteritis. *Curr Gastroenterol Rep* 2014; 16:383. 24604730 10.1007/s11894-014-0383-3

[6] Lee S Park H Ha SY CDK4 amplification predicts recurrence of well-differentiated liposarcoma of the abdomen. *PLoS One* 2014; 9:e99452. 25121597 10.1371/journal.pone.0099452 PMC4133208

[7] Clark MA Fisher C Judson I Soft-tissue sarcomas in adults. *N Engl J Med* 2005; 353:701–711. 16107623 10.1056/NEJMra041866

[8] Herrera-Gomez A Ortega-Gutierrez C Betancourt AM Giant retroperitoneal liposarcoma. *World J Surg Oncol* 2008; 6:115. 18976464 10.1186/1477-7819-6-115 PMC2644689

[9] Bonvalot S Rivoire M Castaing M Primary retroperitoneal sarcomas: a multivariate analysis of surgical factors associated with local control. *J Clin Oncol* 2009; 27:31–37. 19047280 10.1200/JCO.2008.18.0802

[10] Strauss DC Hayes AJ Thway K Surgical management of primary retroperitoneal sarcoma. *Br J Surg* 2010; 97:698–706. 20306527 10.1002/bjs.6994

[11] Kim HS Lee J Yi SY Liposarcoma: exploration of clinical prognostic factors for risk based stratification of therapy. *BMC Cancer* 2009; 9:205. 19558664 10.1186/1471-2407-9-205 PMC2711972

[12] An JY Heo JS Noh JH Primary malignant retroperitoneal tumors: analysis of a single institutional experience. *Eur J Surg Oncol* 2007; 33:376–382. 17129700 10.1016/j.ejso.2006.10.019

[13] Pisters PW Resection of some—but not all—clinically uninvolved adjacent viscera as part of surgery for retroperitoneal soft tissue sarcomas. *J Clin Oncol* 2009; 27:6–8. 19047279 10.1200/JCO.2008.18.7138

[14] Kim EY Kim SJ Choi D Recurrence of retroperitoneal liposarcoma: imaging findings and growth rates at follow-up CT. *AJR Am J Roentgenol* 2008; 191:1841–1846. 19020257 10.2214/AJR.07.3746

[15] Pierie JP Betensky RA Choudry U Outcomes in a series of 103 retroperitoneal sarcomas. *Eur J Surg Oncol* 2006; 32:1235–1241. 16919908 10.1016/j.ejso.2006.07.002

[16] Tseng WW Madewell JE Wei W Locoregional disease patterns in well-differentiated and dedifferentiated retroperitoneal liposarcoma: implications for the extent of resection? *Ann Surg Oncol* 2014; 21:2136–2143. 24705628 10.1245/s10434-014-3643-4

[17] Zhou Z McDade TP Simons JP Surgery and radiotherapy for retroperitoneal and abdominal sarcoma: both necessary and sufficient. *Arch Surg* 2010; 145:426–431. 20479339 10.1001/archsurg.2010.70

[18] Fein DA Corn BW Lanciano RM Management of retroperitoneal sarcomas: does dose escalation impact on locoregional control? *Int J Radiat Oncol Biol Phys* 1995; 31:129–134. 7995743 10.1016/0360-3016(94)E0302-Z

[19] Stoeckle E Coindre JM Bonvalot S Prognostic factors in retroperitoneal sarcoma: a multivariate analysis of a series of 165 patients of the French Cancer Center Federation Sarcoma Group. *Cancer* 2001; 92:359–368. 11466691 10.1002/1097-0142(20010715)92:2<359::aid-cncr1331>3.0.co;2-y

[20] Coia LR Myerson RJ Tepper JE Late effects of radiation therapy on the gastrointestinal tract. *Int J Radiat Oncol Biol Phys* 1995; 31:1213–1236. 7713784 10.1016/0360-3016(94)00419-L

[21] Pawlik TM Ahuja N Herman JM The role of radiation in retroperitoneal sarcomas: a surgical perspective. *Curr Opin Oncol* 2007; 19:359–366. 17545800 10.1097/CCO.0b013e328122d757

[22] Hoffman JP Sigurdson ER Eisenberg BL Use of saline-filled tissue expanders to protect the small bowel from radiation. *Oncology (Williston Park)* 1998; 12:51–54.discussion 54, 60, 62, passim. 9474587

[23] Delaloye JF Cuttat JF Coucke PA Protection of the small bowel with a silicone tissue expander prosthesis and a polyglycolic acid mesh during radiation therapy for cervical carcinoma. *Br J Obstet Gynaecol* 1994; 101:541–542. 8018647 10.1111/j.1471-0528.1994.tb13159.x

[24] Hong A Stevens G Stephen M Protection of the small bowel during abdominal radiation therapy with a tissue expander prosthesis. *Aust N Z J Surg* 2000; 70:690–692. 10976905 10.1046/j.1440-1622.2000.01911.x

[25] Armstrong JG Harrison LB Dattoli M The use of a prosthetic tissue expander to displace bowel from a brachytherapy implant site. *Int J Radiat Oncol Biol Phys* 1990; 19:1521–1523. 2262376 10.1016/0360-3016(90)90367-s

[26] Hoffman JP Lanciano R Carp NZ Morbidity after intraperitoneal insertion of saline-filled tissue expanders for small bowel exclusion from radiotherapy treatment fields: a prospective four year experience with 34 patients. *Am Surg* 1994; 60:473–482.discussion 482-483. 8010560

